# Genetic diversity of the intimin gene (*eae*) in non-O157 Shiga toxin-producing *Escherichia coli* strains in China

**DOI:** 10.1038/s41598-020-60225-w

**Published:** 2020-02-24

**Authors:** Xi Yang, Hui Sun, Ruyue Fan, Shanshan Fu, Ji Zhang, Andreas Matussek, Yanwen Xiong, Xiangning Bai

**Affiliations:** 1 State Key Laboratory of Infectious Disease Prevention and Control, National Institute for Communicable Disease Control and Prevention, Chinese Center for Disease Control and Prevention, Beijing, China; 2 mEpiLab, New Zealand Food Safety Science & Research Centre, School of Veterinary Science, Massey University, Palmerston North, New Zealand; 3 Division of Clinical Microbiology, Department of Laboratory Medicine, Karolinska Institutet, Huddinge, Sweden; 4 Division of Laboratory Medicine, Oslo University Hospital, Oslo, Norway; 5 Collaborative Innovation Center for Diagnosis and Treatment of Infectious Diseases, Hangzhou, China

**Keywords:** Bacteria, Bacteria, Public health, Public health

## Abstract

Shiga toxin-producing *Escherichia coli* (STEC) is an important foodborne pathogen. The increasing incidence of non-O157 STEC has posed a great risk to public health. Besides the Shiga toxin (Stx), the adherence factor, intimin, coded by *eae* gene plays a critical role in STEC pathogenesis. In this study, we investigated the prevalence and polymorphisms of *eae* gene in non-O157 STEC strains isolated from different sources in China. Among 735 non-O157 STEC strains, *eae* was present in 70 (9.5%) strains. Eighteen different *eae* genotypes were identified in 62 *eae*-positive STEC strains with the nucleotide identities ranging from 86.01% to 99.97%. Among which, seven genotypes were newly identified in this study. The eighteen *eae* genotypes can be categorized into five *eae* subtypes, namely β1, γ1, ε1, ζ3 and θ. Associations between *eae* subtypes/genotypes and serotypes as well as origins of strains were observed in this study. Strains belonging to serotypes O26:H11, O103:H2, O111:H8 are associated with particular *eae* subtypes, i.e., β1, ε1, θ, respectively. Most strains from diarrheal patients (7/9, 77.8%) carried *eae*-β1 subtype, while most isolates from cattle (23/26, 88.5%) carried *eae*-ζ3 subtype. This study demonstrated a genetic diversity of *eae* gene in non-O157 STEC strains from different sources in China.

## Introduction

Shiga toxin-producing *Escherichia coli* (STEC) is a group of food-borne pathogens that can cause non-bloody diarrhea, hemorrhagic colitis (HC), and the fatal hemolytic uremic syndrome (HUS) in humans^1^. It has been estimated that there are more than 470 STEC serotypes, among which O157:H7 serotype is usually associated with more severe clinical outcomes^2,3^. However, non-O157 STEC strains such as O26, O45, O103, O111, O121, and O145 (referred to as the ‘top six’ non-O157 STEC) have been increasingly recognized to cause food poisoning, bloody diarrhea, HUS, and other gastrointestinal illnesses in recent years^4,5^. Domestic and wild animals, including cattle, sheep and goats are considered to be the most important reservoirs of STEC^6^. Human infections mainly occur through ingestion of contaminated food or water, exposure to the environment or direct contact with animals^7^.

Shiga toxin (Stx) is considered to be the primary virulence factor of STEC that is responsible for immunopathologies such as HC and HUS^8^. However, Stx alone is insufficient to cause severe illness without the adherence of bacteria to gut epithelial cells^9^. Multiplex genes that enable STEC strains to attach, colonize, produce and secrete toxin proteins have been identified^9^. In a subset of STEC strains, intimin plays a critical role in intestinal colonization, which is encoded by the *eae* gene that resides on the locus of enterocyte effacement (LEE) pathogenicity island. The LEE island encodes a type III secretion system that is responsible for the attaching and effacing (A/E) lesions on intestinal epithelia^10^. A/E lesions is characterized by the local effacement of microvilli, the tight attachment of bacteria to the eukaryotic surface and the subsequent reorganization of filamentous actin to pedestal-like structures^11^. Intimin is also an important virulence factor of other bacteria, such as enteropathogenic *E. coli* (EPEC), *E. albertii*, and *Citrobacter rodentium*^12^.

The full length of *eae* gene is about 2800 nucleotides. The N-terminal of intimin from different sources is highly conserved, while the C-terminal where cellular binding activity is highly variable^13^. Based on the difference of the C-terminal, at least 30 intimin subtypes have been defined, namely, α1, α2, α8, β1, β2, β3, γ1, γ2, ε1, ε2, ε3, ε4, ξ, z, z3, η, η2, θ, τ, ι1, ι2, κ, λ, μ, ν, υ, ο, π, ρ, and σ^14^. Intimin subtypes are correlated with host specificity and tissue tropism^15^. Intimin subtype β1 appears to be the most frequent among atypical EPEC strains from diarrheal patients and animals in China^16^. Several studies have shown the association between serotypes and specific intimin subtypes^17^. For example, O157:H7 and O145:H28 serotypes are associated with the *eae*-γ1 subtype, whereas O26:H11 often carries *eae*-β1, O103:H2 and O121:H19 harbor *eae*-ε, and O111:H8 harbors *eae*-θ subtype^18^. These serotypes were most frequently reported in global dysentery and HUS cases caused by STEC^5^. Thus, *eae* subtyping would be a valuable tool for risk assessment and prediction of disease outcome. However, there are limited studies of intimin characteristics among other non-O157 serotypes. In this study, we investigated the prevalence of *eae* gene and analyzed *eae* subtypes and polymorphisms in non-O157 STEC strains isolated from diarrheal patients, healthy carriers, animals, and raw meats in China.

## Results

### Prevalence of *eae* in the non-O157 STEC collection

Among 735 non-O157 STEC strains, *eae* was present in 70 (9.5%) strains, which were isolated from cattle (n = 41), yak (n = 2), raw beef (n = 3), raw mutton (n = 1), and diarrheal patients (n = 9). Fourteen *eae*-positive strains were identified from unknown sources. All strains recovered from goat, pig, plateau pika, marmot, Tibetan antelope, water, and healthy carriers were *eae*-negative (Table 1 and Table S1).

**Table 1:** The origin and location of 735 non-O157 STEC isolates used in this study.

Source	Location	Year	No. of isolates	No. of *eae*-positive (%)
Cattle	Shandong, Sichuan, Heilongjiang	2009, 2012, 2015, 2017	154	41 (26.6)
Goat	Henan, Sichuan, Shandong	2009, 2017	156	0 (0)
Pig	Chongqing, Beijing, Guizhou, Shandong, Heilongjiang	2011, 2012, 2013, 2015	135	0 (0)
Yak	Qinghai	2012	128	2 (1.6)
Plateau Pika	Qinghai	2012, 2012, 2015	22	0 (0)
Marmot	Qinghai	2012, 2013	8	0 (0)
Tibetan antelope	Qinghai	2014	5	0 (0)
Raw meats	Beijing, Sichuan	2013, 2014	60	4 (6.7)
Diarrheal patient	Henan, Shenzhen, Shanghai, Sichuan, Beijing	2010, 2012, 2013, 2014, 2016, 2018	31	9 (29.0)
Healthy carrier	Qinghai, Shenzhen	2013, 2014	4	0 (0)
Water	Shandong	2017	1	0 (0)
Unknown	Heilongjiang, Guangxi, others	2014	31	14 (45.2)
Total			735	70 (9.5)

### The diversity and subtypes of *eae* in non-O157 STEC strains

**Figure 1:**
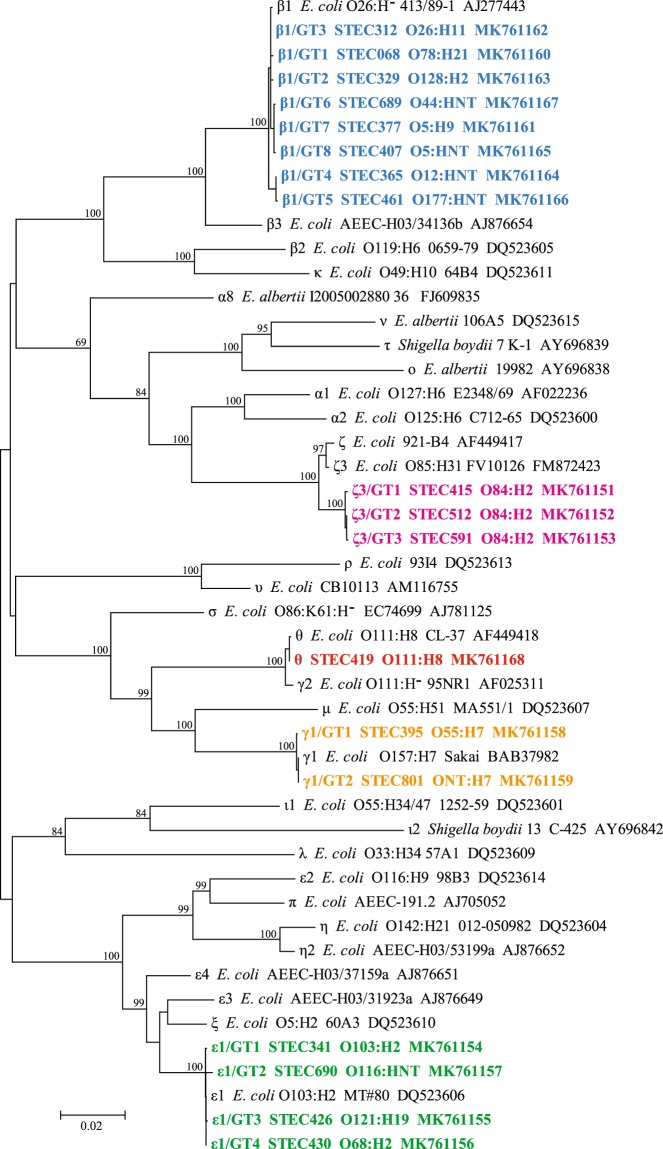
Phylogenetic relationships of 18 different *eae* sequences obtained in this study and 30 *eae* subtypes reference sequences based on Neighbor-Joining method. The corresponding *eae* subtype, strain name, and GenBank accession number are listed on the right. The *eae* subtypes/genotypes identified in this study are indicated in bold and different colors. Scale bar indicates genetic distance.

The complete *eae* sequences were obtained from 62 out of 70 strains, eight strains which failed to yield the complete *eae* sequences were excluded in this study. Among the 62 *eae* sequences, 18 unique *eae* sequences were identified (Table 2), the nucleotide identities among the 18 unique *eae* sequences ranged from 86.01% to 99.97% based on pairwise comparisons. Five *eae* subtypes, namely, β1, γ1, ε1, ζ3 and θ, were assigned based on phylogenic analysis. *eae* sequence polymorphism, designated as genotypes (GTs) were identified in each *eae* subtype to represent the diversity within a subtype. Except θ, the other four subtypes contained 2 to 8 genotypes respectively (Fig. 1). The BLASTn search against GenBank database (nr/nt) showed that 7 *eae* genotypes (β1/GT5, β1/GT6, β1/GT8, ε1/GT1, ζ1/GT2, ζ1/GT2 and ζ1/GT3) in this study are unique comparing with all available *eae* sequences in the database (accessed 25/7/2019).

**Table 2:** *eae* subtypes of 62 *eae*-positive non-O157 STEC strains.

*eae* subtype /genotype	Origin	Serotype	*stx* subtype	Sequence type
β1 /GT1 (2)	Yak	O78:H21 (1), O78:HNT (1)	*stx*_2a_	ST3884 (1), ST40 (1)
β1 /GT2 (1)	Unknown	O128:H2	*stx*_2f_	N3
β1 /GT3 (8)	Diarrheal patient (5), Unknown (3)	O26:H11	*stx*_1a_ (6), *stx*_2a_ (2)	ST21
β1 /GT4 (1)	Raw beef	O12:HNT	*stx*_2c_	ST659
β1 /GT5 (8)	Cattle	O177:HNT	*stx*_2c_	ST659 (1), ST7220 (7)
β1 /GT6 (1)	Cattle	O44:HNT	*stx*_1a_	N1
β1 /GT7 (2)	Diarrheal patient (1), Raw mutton (1)	O5:H9 (1), O5:HNT (1)	*stx*_1a_	ST342
β1 /GT8 (1)	Diarrheal patient	O5:HNT	*stx*_1a_	ST342
ε1 /GT1 (1)	Unknown	O103:H2	*stx*_1a_	N4
ε1 /GT2 (3)	Cattle	O5:HNT (1), O116:HNT (1), ONT:HNT (1)	*stx*_2c_ (2), *stx*_1a+_ *stx*_2c_ (1)	ST119 (2), N2
ε1 /GT3 (1)	Unknown	O121:H19	*stx*_2a_	ST655
ε1 /GT4 (1)	Unknown	O68:H2	*stx*_1a_	N5
γ1 /GT1 (2)	Unknown	O55:H7	*stx*_1a_	ST335
γ1 /GT2 (1)	Diarrheal patient	ONT:H7	*stx*_1a+_ *stx*_2a_	ST11
θ (5)	Raw beef (2), Unknown (3)	O103:H25 (2), O111:H8 (2), O111:HNT (1)	*stx*_1a_	ST16 (3), ST343 (2)
ζ3 /GT1 (1)	Diarrheal patient	O84:H2	*stx*_1a_	ST306
ζ3 /GT2 (22)	Cattle (22)	O84:H2	*stx*_1a_	ST306
ζ3 /GT3 (1)	Cattle (1)	O84:H2	*stx*_1a_	ST306

Three major *eae* genotypes (ζ3/GT2, β1/GT3 and β1/GT5) contained 22, 8 and 8 strains respectively, and 10 genotypes contained only one strain, while the rest contained two to five strains (Table 2).

### *eae* genotypes in correlation with serotypes

Fifteen different O serogroups and 9 different H types were identified among the 62 STEC strains, which belonged to 19 serotypes: ONT:H7, O103:H25, O103:H2, O111:HNT, O111:H8, O116:HNT, O12:HNT, O121:H19, O128:H2, O117:HNT, O26:H11, O44:HNT, O5:HNT, O5:H9, O55:H7, O68:H2, O78:HNT, O78:H21 and O84:H2. Two and 17 strains were O and H untypable, respectively. The most predominant serotype was O84:H2 (24/62, 38.7%), followed by O26:H11 (8/62, 12.9%), and O177: HNT (7/62, 11.3%).

A link was observed between serotypes and *eae* genotypes. Each *eae* genotype contained one to three different serotypes, with the exception of serotypes O5:HNT and O84:H2. Strains of O5:HNT serotype were assigned to β1/GT7, β1/GT8 or ε1/GT10 *eae* genotype, while O84:H2 strains carried *eae* ζ3/GT1, ζ3/GT2, or ζ3/GT3 genotypes (Table 2).

### *stx* type/subtypes in *eae* positive non-O157 STEC strains

Overall, 43 strains harbored *stx*_1_ only, 17 strains contained *stx*_2_ only and 2 strains possessed both *stx*_1_ and *stx*_2_. Only one *stx*_1_ subtype, *stx*_1a_, was identified, while three *stx*_2_ subtypes (*stx*_2a_, *stx*_2c_ and *stx*_2f_) were detected among the 62 STEC strains.

Among 24 *eae*-β1 harboring strains, 10 strains carried *stx*_1a,_ nine carried *stx*_2c_ and the other possessed *stx*_2a_, or *stx*_2f_ subtypes. The strains with *eae*-ε1 subtype carried diverse *stx* subtypes: two strains contained *stx*_1a_, two strains carried *stx*_2c_, one carried *stx*_2a_, and one harbored both *stx*_1a_ and *stx*_2c_. Two of *eae*-γ1 strains carried *stx*_1a_ and one harbored both *stx*_1a_ and *stx*_2a_. All the 24 *eae*-ζ3 and 5 *eae*-θ containing strains possessed *stx*_1a_ (Table 2).

### STEC origin correlated with the *eae* genotypes

STEC strains carrying *eae*-β1 subtype were detected from all sources investigated in this study. A concordance was observed between STEC origin and *eae* genotypes. Each *eae* genotype contained strains from a specific host source with the exception of three genotypes. The β1/GT7 genotype was detected from strains isolated from human and mutton. The β1/GT3 or θ genotype contain strains from two different sources (Table 2).

Human-derived strains belonged to five *eae* genotypes, among which, three (β1/GT8, γ1/GT2, and ζ1/GT3) are unique. Furthermore, all cattle-derived strains belonged to five unique *eae* genotypes, i.e. β1/GT5, β1 /GT6, ε1/GT2, ζ3/GT2, and ζ3/GT3 (Table 2).

### MLST analysis of *eae*-positive non-O157 STEC

The 62 strains were typed into 18 sequence types (STs) with 5 novel STs named as N1-N5. One new ST (strain STEC430) was resulted from a novel allele in *icd*. The other four new STs (strain STEC329, STEC341, STEC689, STEC790) were due to the new combinations of previously known alleles. An *eae* genotype was corresponding to one or more STs and vice versa (Table 2).

**Figure 2:**
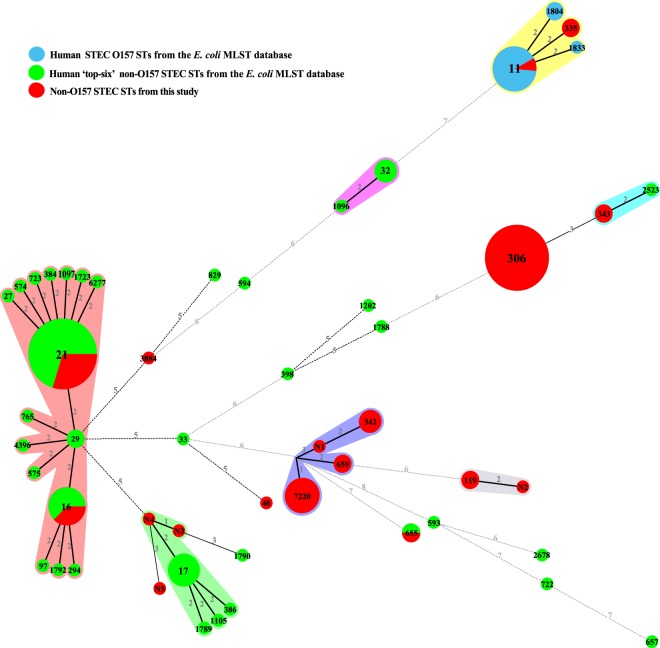
Minimum spanning tree of 62 STs from this study (red), 13 STs from human O157 STEC (blue), and 65 STs from human ‘top six’ non-O157 STEC (green). Each circle represents a ST, with the pie divided proportionally to the number of isolates in that ST from different sources. The number in a circle indicates the ST number. The numbers on connecting lines represent the number of allelic differences between two STs.

A minimum spanning tree was constructed with the STs from this study and those of O157 and ‘top six’ non-O157 STEC from MLST database (Fig. 2). Most STs differed from each other by 2 or more alleles, while two pairs of STs (N1 and ST7220, N3 and N4) differed from each other by only 1 allele. STs of human O157 STEC are different from those of ‘top six’ non-O157 STEC. Only three STs (ST16, ST21 and ST655) were shared by strains from this study and human ‘top six’ non-O157 STEC assigned as O111, O26 and O121 serogroups, respectively. However, STs of O103 serogroup are diverse, which were different from those of MLST database. An ONT:H7 strain obtained in this study was typed as ST11, which is often recognized as ST of STEC O157:H7 (Table 2 and Table S1).

## Discussion

Intimin encoded by *eae* is an important virulence factor in many STEC strains, which plays a critical role in intestinal colonization. Previous studies revealed that most clinical STEC strains possessed *eae*^19,20^. Moreover, the presence of *eae* was significantly associated with a higher risk for HUS development^21^. STEC O157 isolates were strongly associated with the simultaneous presence of both *stx*_2_ and *eae*, forming the basis of why STEC O157 predominates in patients with HUS, when compared with non-O157 strains^22^. However, a subsequent study reported that *eae* was detected in the majority (52.5%) of non-O157 STEC strains in England^23^. A recent investigation of STEC infections in the south east of England revealed that 76% of non-O157 HUS-associated STEC isolates possessed *eae* gene^24^. These data indicated that the presence of *eae* is strongly related with disease severity irrespective of seropathotypes. In this study, we observed a lower prevalence of *eae* (29.0%) among non-O157 STEC strains from diarrheal patients, which might partially be due to limitation of the current study where the source of 14 *eae*-positive isolates is unavailable. On the other hand, the low prevalence of *eae* in diarrheal patients could account for the less severe disease in this study. Among the nine *eae*-positive isolates from diarrheal patients, only one was both *stx*_1a_ and *stx*_2a_ positive, while the rest all harbored *stx*_1a_ only (Table 2). Stx2 positive strain especially Stx2a induces more severe clinical symptoms and higher mortality than other Stx subtypes^25^. Previous study reported that 20% of meat STEC isolates carried *eae*^19^, 36% of the cattle isolates possessed *eae*^26^. In this study, we found that 6.7% of raw meat isolates possessed *eae*, the prevalence of *eae* in cattle isolates is 26.6%. The variation of *eae* prevalence among different studies possibly due to several factors including sample sources, isolation/detections assays, or geographic distribution.

The *eae* gene was classified into different subtypes based on the variety of the 280 amino acids C-terminal region^13^. The highly divergent C-terminal region of *eae* constitutes the molecule that binding to receptors on the epithelial cell^27^. Various *eae* subtypes may confer distinct colonization patterns within the human intestine, thus leading to distinct pathogenic capacity. Among the known *eae* subtypes described so far, four subtypes (β, ε, γ1, θ) have been reported to be associated with more virulent STEC and thus posing greater health risk^28^. In a previous study, the *eae* subtypes of STEC strains recovered from children with HUS in Uruguay, include γ1, γ2 and β1^29^, highlighting the clinical significance of these *eae* subtypes. In this study, we found that β1 and ζ3 were the most prevalent *eae* subtypes, which were detected in strains from different sources. Notably, *eae*-β1 was the most predominant subtype among strains from diarrheal patients^30^.

Studies have indicated association between serotypes and *eae* subtypes in STEC strains, particularly predominant serotypes. Strains belonging to serotypes O157:H7, O26:H11, O103:H2, O111:H8, and O145:H28 are associated with particular *eae* subtypes, i.e., γ1, β1, ε, θ, and γ1, respectively^31,32^. Consistent with previous studies, we found that all eight O26:H11 strains (five from diarrheal patients and three from unknown origin) in this study carried the *eae*-β1 subtype, all three O111 strains harbored the *eae*-θ subtype, and one O103 strain possessed the *eae*-ε1 subtype. However, the origin of three O111 strains and one O103 strain were unavailable. Notably, the three O111 strains share identical *eae* sequences with the outbreak strain O111:H8 in the United States^33^.The *eae* sequence of strain STEC801 (ONT:H7) isolated from a diarrheal patient was identical to that of strain Sakai, which caused a notorious outbreak in Japan in 1996^34^. Comparison with STs observed in human non-O157 STEC infections gives an indication of the potential risk for different *eae* subtype STEC. Strains of the three STs (ST16, ST21 and ST655) in this study had the same O serogroups with ‘top six’ non-O157 STEC (O111, O26 and O121). The three *eae* subtypes were θ, β1 and ε1, respectively. These results indicated that determination of *eae* subtypes could be used as a valuable tool in combinations with serotypes, and other virulence factors in risk assessment and prediction of severe disease outcomes.

Shiga toxin subtypes have been found to differ in toxin potency. Strains that carry Stx2a (with or without Stx2c) are often associated with severe symptoms such as HC and HUS^35,36^. The *stx*_2a_ gene is most often present in *eae* positive STEC strains and has consistently been associated with HUS^37^. In this study, 6 out of 62 isolates carried *stx*_2a_ subtypes, which were isolated from diarrheal patients, yak and other unknown sources. Notably, the 6 *stx*_2a_ isolates harbored *eae-*β1, *eae-*ε1 and *eae-*γ1 subtypes, all of which were associated with high virulence, thus they were likely to have high pathogenic potential to humans.

Besides intimin gene *eae*, the plasmid-carried enterohemolysin gene (*ehxA*) also plays an important role in STEC pathogenicity and frequently associated with diarrheal disease and HUS^38^. In a previous study, we described the presence and genetic diversity of the *ehxA* gene in 434 non-O157 STEC isolates. The *ehxA* gene was positive in 138 (31.8%) isolates, and 15 (10.9%) *ehxA*-positive isolates harbored *eae*, which were grouped into *ehxA* group I (n = 2) and group II (n = 13). All strains from diarrheal patients belonged to *ehxA* group II and most of those strains harbored *eae*. Thus, *ehxA* group II and *eae*-positive strains were clinically related^39^.

In this study, we observed the association between *eae* genotypes and host sources. Most strains from diarrheal patients (7/9, 77.8%) carried *eae*-β1 subtype, and most isolates from cattle (23/26, 88.5%) carried *eae*-ζ3 subtype. However, the prevalence of *eae* subtypes in a specific source varied significantly among studies. For instance, Tostes *et al*.^28^ reported the predominance of *eae* subtypes *λ/* γ1 and β in cattle isolates, and *λ/* γ1 in human isolates. Similarly, Blanco *et al*.^39^ reported *eae*-ζ as most frequent subtype in *E. coli* isolates from sheep in Spain. Whereas, Aktan *et al*.^40^ identified *eae*-β and *eae*-γ as the most frequent subtypes among *E. coli* from sheep in England and Wales. The distribution of *eae* subtypes in the same source may vary among different regions.

In conclusion, the current study reports the prevalence and subtype of *eae* gene among non-O157 STEC strains from a wide variety of sources in China. Among 735 non-O157 STEC strains, *eae* was present in 70 (9.5%) strains isolated from diarrheal patients, animals, raw meats and other unknown sources. Five *eae* subtypes and 18 different *eae* genotypes were identified, suggesting the high diversity of *eae* among different sources. To our knowledge, this is the first study investigating the prevalence of *eae* subtypes among non-O157 STEC strains from a wide range of sources. Our study suggests associations between *eae* subtypes and serotypes as well as host sources. Furthermore, it can be inferred from this study that the determination of *eae* subtype could be considered together with seropathotypes and other virulence factors in risk assessment of STEC infections.

## Materials and Methods

### Ethics statement

The current study and all experimental protocols were approved by the ethic committee of the National Institute for Communicable Diseases Control and Prevention, China CDC, with the number ICDC-2017006. This work was part of STEC surveillance program conducted in China, the informed permission has been obtained from patients and the owners of animals to use fecal samples and conduct relevant studies. All methods used in this study were performed in accordance with the relevant guidelines and regulations.

### Bacterial strains and detection of *eae* gene

A total of 735 non-O157 STEC strains collected during April 2009 to December 2018 were used in this study. Most strains were isolated from different samples through the STEC surveillance conducted in China. Others were obtained from local Centers for Disease Control and Prevention (CDC) in eleven geographical regions in China. Some strains were reported in our previous studies^41–43^. Of the 735 STEC strains, 608, 60, 35, and 1 were isolated from animals, raw meats, humans and environmental water, respectively. The sources of 31 strains were unavailable (Table 1 and Table S1). All strains were confirmed to be STEC by using previously described methods^41^.

The presence of *eae* gene among all 735 non-O157 STEC strains were screened using PCR method with primer *eae-*F (5′-TCAATGCAGTTCCGTTATCAGTT-3′) and *eae*-R (5′-GTAAAGTCCGTTACCCCAACCTG-3′)^44^.

### Serotyping and *stx* subtyping

The O serogroup of each isolate was initially screened by an O-genotyping PCR method designed by Iguchi *et al*.^45^, and further confirmed by using *E. coli* antisera, O1–O188 (Statens Serum Institut, Hillerød, Denmark). The entire coding sequence of *fliC* was amplified by PCR with the primers fliC-F (5′-ATGGCACAAGTCATTAATACCCAAC-3′) and fliC-R (5′-CTAACCCTGCAGCAGAGACA-3′) as reported by Fields *et al*. and then sequenced with Sanger sequencing^46^. The sequences were analyzed with the SerotypeFinder database (https://cge.cbs.dtu.dk/services/SerotypeFinder/) to determine H types^47^.

The *stx*_1_ subtypes (*stx*_1a_*, stx*_1c_, *stx*_1d_) and *stx*_2_ subtypes (*stx*_2a_ to *stx*_2h_) were determined by a PCR-based subtyping protocol in combination with a phylogeny scheme as described previously^48,49^.

### Sequencing of the complete *eae* gene

The complete *eae* gene was obtained by PCR using previously described method^16^. Two additional primer designed in this study were used for sequencing: *eae*R3-A (5′-TCCATGTGTATTTTCCATTGCC-3′) and *eae*R3-B (5′-TATATTTCCCATCACCTCCAC-3′). All PCR products were visualized by agarose gel electrophoresis and purified by using a QIAquick PCR purification kit (Qiagen, Germany), and then sequenced using BigDye™ Terminator V3.1 Cycle Sequencing kit (Applied Biosystems, USA).

### *eae* subtyping and polymorphism analysis

Each of the sequenced ~3.2 kb LEE region that contained the complete *eae* gene was checked and assembled by SeqMan II (DNASTAR Inc., USA). The *eae* subtypes reference sequences were downloaded from GenBank. The MEGA 7 software (www.megasoftware.net)^50^ was used to align the complete *eae* sequences obtained in this study and the reference *eae* sequences. A Neighbor-Joining tree was constructed with maximum composite likelihood model. Bootstrap analyses were performed (1,000 replicates) to estimate the stability and genetic distances were calculated by the maximum composite likelihood method. A novel subtype was defined by a cutoff value of 95% nucleotide sequence identity as described previously^51^.

### Multilocus sequence typing (MLST)

Seven housekeeping genes (*adk*, *fumC*, *gyrB*, *icdF*, *mdh*, *purA*, and *recA*) were amplified by PCR and sequenced according to the scheme provided by the *E. coli* MLST website (http://enterobase.warwick.ac.uk/species/ecoli/allele_st_search). Each locus was assigned an allele number by comparing sequences against the *E. coli* MLST database. The allelic profile of seven housekeeping genes was used to generate a specific sequence type (ST) for each isolate. STs of human STEC O157 and the ‘top six’ non-O157 serogroups were downloaded from the *E. coli* MLST database for comparison. A minimum spanning tree was generated with BioNumerics software version 4.0 (Applied Maths, Belgium).

### Nucleotide sequence accession numbers

The 18 diverse *eae* sequences obtained in this study were submitted to GenBank (Acc. MK761151–MK761168).

## Supplementary information

Supplementary Information.
