# Supplementary material for: Genetic diversity of the intimin gene (eae) in non-O157 Shiga toxin-producing Escherichia coli strains in China
Source: Sci Rep. 2020 Feb 24;10:3275. doi: 10.1038/s41598-020-60225-w (PMC7040016; doi:10.1038/s41598-020-60225-w)
Supplement: Supplementary file 1 — Supplementary Information. [file 41598_2020_60225_MOESM1_ESM.pdf]

Supplementary Information

Genetic diversity of the intimin gene (*eae*) in non-O157 Shiga toxin-producing *Escherichia coli* strains in China

Xi Yang [1], Hui Sun [1], Ruyue Fan [1], Shanshan Fu [1], Ji Zhang [2], Andreas Matussek [3], Yanwen Xiong [1,4], Xiangning Bai [1,3*]

[1] State Key Laboratory of Infectious Disease Prevention and Control, National Institute for Communicable Disease Control and Prevention, Chinese Center for Disease Control and Prevention, Beijing, China.

[2] mEpiLab, New Zealand Food Safety Science & Research Centre, School of Veterinary Science, Massey University, New Zealand.

[3] Division of Clinical Microbiology, Department of Laboratory Medicine, Karolinska Institutet, Karolinska University Hospital, Huddinge, Sweden.

[4] Collaborative Innovation Center for Diagnosis and Treatment of Infectious Diseases, Hangzhou, China.

*Corresponding author. Email: baixiangning@icdc.cn

Table S1. Characteristics of 735 non-O157 STEC strains used in this study

Strain	Origin	Location	Year	O-serogroup	H-type	Virulence genes		
						stx 1	*stx* 2	*eae*
STEC003	Goat	Henan	2009	O45	H2	1c	2d	-
STEC004	Goat	Henan	2009	O118	H16	-	2d	-
STEC005	Goat	Henan	2009	O39	H19	-	2e	-
STEC006	Goat	Henan	2009	O45	H2	1c	2d	-
STEC007	Goat	Henan	2009	O91	H14	1c	2d	-
STEC008	Goat	Henan	2009	O21	H25	-	2d	-
STEC009	Goat	Henan	2009	O91	H14	1c	2d	-
STEC010	Goat	Henan	2009	O110	H16	1a	2d	-
STEC011	Goat	Henan	2009	O103	H8	1c	-	-
STEC012	Goat	Henan	2009	O21	H25	1a	-	-
STEC013	Goat	Henan	2009	O21	H25	1a	-	-
STEC014	Goat	Henan	2009	O21	H25	1a	-	-
STEC015	Goat	Henan	2009	O103	H8	1c	-	-
STEC016	Goat	Henan	2009	O103	H8	1c	-	-
STEC017	Goat	Henan	2009	O141ac	H25	-	2g	-
STEC018	Goat	Henan	2009	O15	H21	1a	-	-
STEC019	Goat	Henan	2009	O12	H21	1a	-	-
STEC020	Goat	Henan	2009	O12	H21	1a	-	-
STEC021	Goat	Henan	2009	O21	H25	1a	-	-
STEC022	Goat	Henan	2009	O21	H25	1a	-	-
STEC023	Goat	Henan	2009	O103	H8	1c	-	-
STEC024	Goat	Henan	2009	O103	H8	1c	-	-
STEC025	Goat	Henan	2009	O103	H8	1c	-	-
STEC026	Goat	Henan	2009	O103	H8	1c	-	-
STEC027	Goat	Henan	2009	O103	H8	1c	-	-
STEC028	Goat	Henan	2009	OUT	H21	1c	-	-
STEC029	Goat	Henan	2009	O15	H21	1c	-	-
STEC030	Goat	Henan	2009	OUT	H21	1a	-	-
STEC031	Diarrheal patient	Henan	2011	O107	H7	1a	-	-
STEC032	Diarrheal patient	Henan	2011	O107	H7	1a	-	-
STEC033	Beef cattle	Heilongjiang	2009	O113	H19	1a	2a	-
STEC036	Yak	Qinghai	2012	NT	H21	-	2d	-
STEC037	Yak	Qinghai	2012	NT	H44	1a	-	-
STEC038	Yak	Qinghai	2012	O8	H9	1a	2d	-
STEC039	Yak	Qinghai	2012	NT	H7	1a	-	-
STEC040	Yak	Qinghai	2012	O6	H21	1a	-	-
STEC041	Yak	Qinghai	2012	O6	H21	1a	-	-
STEC042	Yak	Qinghai	2012	O78	H45	-	2g	-
STEC043	Yak	Qinghai	2012	O8	H9	1a	2d	-
STEC044	Yak	Qinghai	2012	O8	H16	-	2b	-
STEC045	Yak	Qinghai	2012	O8	H9	1a	2d	-
STEC046	Yak	Qinghai	2012	O6	NT	1a	-	-
STEC047 -1	Yak	Qinghai	2012	O165	H21	1a	-	-
STEC047 -2	Yak	Qinghai	2012	O78	H45	-	2g	-
STEC048	Yak	Qinghai	2012	O78	H45	-	2g	-
STEC049	Yak	Qinghai	2012	NT	NT	-	2c	-
STEC050	Yak	Qinghai	2012	O8	H19	1a	-	-
STEC051	Yak	Qinghai	2012	NT	H21	1a	-	-
STEC052	Yak	Qinghai	2012	NT	H21	1a	-	-

STEC053	Yak	Qinghai	2012	NT	NT	1a	2a+2b	-
STEC054	Yak	Qinghai	2012	NT	H8	1a	2a+2b	-
STEC055	Yak	Qinghai	2012	O66	H8	1a	-	-
STEC056	Yak	Qinghai	2012	O78	NT	-	2a	+
STEC057	Yak	Qinghai	2012	O78	H21	-	2c	-
STEC058	Yak	Qinghai	2012	O22	H8	-	2b	-
STEC059	Yak	Qinghai	2012	O12/O78	H44	-	2d	-
STEC060	Yak	Qinghai	2012	O117	H21	-	2b	-
STEC061	Yak	Qinghai	2012	O78	H45	-	2g	-
STEC062	Yak	Qinghai	2012	O8	H45	-	2g	-
STEC063	Yak	Qinghai	2012	O8	H19	1a	-	-
STEC064	Yak	Qinghai	2012	O8	H19	1a	-	-
STEC065	Yak	Qinghai	2012	O8	H9	1a	2d	-
STEC066	Yak	Qinghai	2012	O8	H9	1a	2d	-
STEC067	Yak	Qinghai	2012	O8	H16	-	2b	-
STEC068	Yak	Qinghai	2012	O78	H21	-	2a	+
STEC069	Yak	Qinghai	2012	O52	H2	-	2b	-
STEC070	Yak	Qinghai	2012	O78	H45	-	2g	-
STEC071	Yak	Qinghai	2012	O8	H9	1a	2d	-
STEC072	Yak	Qinghai	2012	O8	H9	1a	2d	-
STEC073	Yak	Qinghai	2012	O8	H9	1a	2d	-
STEC074	Yak	Qinghai	2012	O117	H21	-	2b	-
STEC075	Yak	Qinghai	2012	O66	H21	-	2a+2b	-
STEC076	Yak	Qinghai	2012	NT	H8	-	2b	-
STEC077	Yak	Qinghai	2012	O158	H8	-	2b	-
STEC078	Yak	Qinghai	2012	O158	H8	-	2b	-
STEC079	Yak	Qinghai	2012	O2	H45	1a	-	-
STEC080	Yak	Qinghai	2012	O2	H45	1a	-	-
STEC081	Yak	Qinghai	2012	O78	H8	-	2a	-
STEC082	Yak	Qinghai	2012	O78	H8	-	2a	-
STEC083	Yak	Qinghai	2012	O2	H45	1a	-	-
STEC084	Yak	Qinghai	2012	O66	H21	-	2a+2b	-
STEC085	Yak	Qinghai	2012	O8	H16	-	2b	-
STEC086	Yak	Qinghai	2012	O8	H45	-	2b	-
STEC087	Yak	Qinghai	2012	O8	H45	-	2b	-
STEC088	Yak	Qinghai	2012	O22	H8	-	2b	-
STEC089	Yak	Qinghai	2012	NT	H21	1a	-	-
STEC090	Yak	Qinghai	2012	O22	H8	-	2b	-
STEC091	Yak	Qinghai	2012	NT	H21	1a	-	-
STEC092	Yak	Qinghai	2012	NT	H8	-	2b	-
STEC093	Yak	Qinghai	2012	O2	H45	1a	-	-
STEC094	Yak	Qinghai	2012	O137	H21	1a	2a	-
STEC095	Yak	Qinghai	2012	NT	H8	1a	-	-
STEC096	Yak	Qinghai	2012	O127	H8	1a	2d	-
STEC097	Yak	Qinghai	2012	O2	H45	1a	-	-
STEC098	Yak	Qinghai	2012	O117	H21	-	2b	-
STEC099	Yak	Qinghai	2012	O117	H21	-	2b	-
STEC100	Yak	Qinghai	2012	NT	H8	1a	2b	-
STEC101	Yak	Qinghai	2012	O8	H2	-	2d	-
STEC102	Yak	Qinghai	2012	O165	H8	1a	-	-
STEC103	Yak	Qinghai	2012	O165	H8	1a	-	-
STEC104	Yak	Qinghai	2012	O8	H16	-	2b	-
STEC105	Yak	Qinghai	2012	O8	H16	-	2b	-
STEC106	Yak	Qinghai	2012	O117	H2	-	2b	-

STEC107	Yak	Qinghai	2012	O117	H2	-	2b	-
STEC108	Yak	Qinghai	2012	O117	H2	-	2b	-
STEC109	Yak	Qinghai	2012	O117	H2	-	2b	-
STEC110	Yak	Qinghai	2012	O8	H16	-	2b	-
STEC111	Yak	Qinghai	2012	O8	H16	-	2b	-
STEC112	Yak	Qinghai	2012	O117	H21	-	2b	-
STEC113	Yak	Qinghai	2012	O117	H21	-	2b	-
STEC114	Yak	Qinghai	2012	O117	H21	-	2b	-
STEC115	Yak	Qinghai	2012	O6	H14	-	2b	-
STEC116	Yak	Qinghai	2012	O6	H14	-	2b	-
STEC117	Yak	Qinghai	2012	O158	H16	-	2b	-
STEC118	Yak	Qinghai	2012	O8	H16	-	2b	-
STEC119	Yak	Qinghai	2012	NT	H8	1a	2d	-
STEC120	Yak	Qinghai	2012	O165	H8	1a	2d	-
STEC121	Yak	Qinghai	2012	O117	H21	-	2b	-
STEC122	Yak	Qinghai	2012	NT	H40	-	2b	-
STEC123	Yak	Qinghai	2012	O117	H21	-	2b	-
STEC124	Yak	Qinghai	2012	O117	H21	-	2b	-
STEC125	Yak	Qinghai	2012	O8	H16	-	2b	-
STEC126	Yak	Qinghai	2012	O117	H21	-	2b	-
STEC127	Yak	Qinghai	2012	O78	H8	1a	2d	-
STEC128	Yak	Qinghai	2012	O78	H8	1a	2d	-
STEC129	Yak	Qinghai	2012	NT	H21	1a	2d	-
STEC130	Yak	Qinghai	2012	NT	H21	1a	2d	-
STEC131	Yak	Qinghai	2012	NT	H44	1a	-	-
STEC132	Yak	Qinghai	2012	O123	H8	1a	2b	-
STEC133	Yak	Qinghai	2012	O8	H16	-	2b	-
STEC134	Yak	Qinghai	2012	O8	H16	-	2b	-
STEC135	Yak	Qinghai	2012	O8	H16	-	2b	-
STEC136	Yak	Qinghai	2012	O8	H16	-	2b	-
STEC137	Yak	Qinghai	2012	O8	H16	-	2b	-
STEC138	Yak	Qinghai	2012	O149	H45	-	2d	-
STEC139	Yak	Qinghai	2012	O149	H45	-	2d	-
STEC140	Yak	Qinghai	2012	NT	H8	-	2a	-
STEC141	Yak	Qinghai	2012	NT	H8	-	2a	-
STEC142	Yak	Qinghai	2012	O2	H45	1a	-	-
STEC143	Yak	Qinghai	2012	O2	H45	1a	-	-
STEC144	Yak	Qinghai	2012	O78	H45	-	2a+2c	-
STEC145	Yak	Qinghai	2012	O78	H21	-	2a+2c	-
STEC146	Yak	Qinghai	2012	O2	H21	-	2a+2c	-
STEC147	Yak	Qinghai	2012	O2	H45	1a	-	-
STEC148	Yak	Qinghai	2012	O22	H8	-	2b	-
STEC149	Yak	Qinghai	2012	O2	H45	1a	-	-
STEC150	Yak	Qinghai	2012	O2	H45	1a	-	-
STEC151	Yak	Qinghai	2012	O2	H45	1a	-	-
STEC152	Yak	Qinghai	2012	O78	H8	-	2a	-
STEC153	Yak	Qinghai	2012	O78	H8	-	2a	-
STEC154	Yak	Qinghai	2012	O78	H44	-	2d	-
STEC155	Yak	Qinghai	2012	O12	H12	1a	-	-
STEC156	Yak	Qinghai	2012	O78	H8	-	2a	-
STEC157	Yak	Qinghai	2012	O2	H45	1a	-	-
STEC158	Yak	Qinghai	2012	O78	H8	-	2a+2c	-
STEC159	Yak	Qinghai	2012	O2	H45	1a	-	-
STEC160	Yak	Qinghai	2012	O2	H45	1a	-	-

STEC161	Yak	Qinghai	2012	O52	H2	-	2b	-
STEC162	Yak	Qinghai	2012	O52	H2	-	2b	-
STEC163	Pig	Chongqing	2012	O2	H32	-	2e	-
STEC164	Pig	Chongqing	2012	O2	H32	-	2e	-
STEC165	Pig	Chongqing	2012	O2	H32	-	2e	-
STEC166	Pig	Chongqing	2012	O2	H32	-	2e	-
STEC167	Pig	Chongqing	2012	O2	H32	-	2e	-
STEC168	Pig	Chongqing	2012	O2	H32	-	2e	-
STEC169	Pig	Chongqing	2012	O2	H32	-	2e	-
STEC170	Pig	Chongqing	2012	O2	H32	-	2e	-
STEC171	Pig	Chongqing	2012	O116	H11	-	2e	-
STEC172	Pig	Chongqing	2012	O2	H32	-	2e	-
STEC173	Pig	Chongqing	2012	O20	H30	-	2e	-
STEC174	Pig	Chongqing	2012	O20	H26	-	2e	-
STEC175	Pig	Chongqing	2012	O20	H30	-	2e	-
STEC176	Pig	Chongqing	2012	O76	H25	-	2e	-
STEC177	Pig	Chongqing	2012	O20	H26	-	2e	-
STEC178	Pig	Chongqing	2012	O20	H30	-	2e	-
STEC179	Pig	Chongqing	2012	O20	H30	-	2e	-
STEC180	Pig	Chongqing	2012	NT	H30	-	2e	-
STEC181	Pig	Chongqing	2012	NT	H30	-	2e	-
STEC182	Pig	Chongqing	2012	O114	H30	-	2e	-
STEC183	Pig	Chongqing	2012	O65	H30	-	2e	-
STEC184	Pig	Chongqing	2012	O100	H20	-	2e	-
STEC185	Pig	Chongqing	2012	O100	H20	-	2e	-
STEC186	Pig	Chongqing	2012	O20	H30	-	2e	-
STEC187	Pig	Chongqing	2012	O20	H30	-	2e	-
STEC188	Pig	Beijing	2012	NT	H21	-	2e	-
STEC189	Pig	Beijing	2012	NT	H21	-	2e	-
STEC190	Pig	Beijing	2012	NT	H33	-	2e	-
STEC191	Pig	Beijing	2012	NT	H30	-	2e	-
STEC192	Pig	Beijing	2012	O2	H32	-	2e	-
STEC193	Pig	Beijing	2012	O2	H32	-	2e	-
STEC194	Pig	Beijing	2012	NT	H30	-	2e	-
STEC195	Pig	Beijing	2012	NT	H30	-	2e	-
STEC196	Pig	Beijing	2012	O2	H30	-	2e	-
STEC197	Pig	Beijing	2012	O172	H30	-	2e	-
STEC198	Pig	Beijing	2012	O172	H30	-	2e	-
STEC199	Pig	Beijing	2012	O20	H30	-	2e	-
STEC200	Pig	Beijing	2012	O20	H30	-	2e	-
STEC201	Pig	Beijing	2012	O20	H30	-	2e	-
STEC202	Pig	Beijing	2012	O20	H30	-	2e	-
STEC203	Pig	Beijing	2012	NT	H30	-	2e	-
STEC204	Pig	Beijing	2012	O20	H30	-	2e	-
STEC205	Pig	Beijing	2012	O20	H30	-	2e	-
STEC206	Pig	Beijing	2012	O20	H30	-	2e	-
STEC207	Pig	Beijing	2012	O20	H30	-	2e	-
STEC208	Pig	Beijing	2012	NT	H30	-	2e	-
STEC209	Pig	Beijing	2012	NT	H30	-	2e	-
STEC210	Pig	Beijing	2012	O7	H30	-	2e	-
STEC211	Pig	Beijing	2012	NT	H19	-	2e	-
STEC212	Pig	Beijing	2012	NT	H19	-	2e	-
STEC213	Pig	Beijing	2012	NT	H19	-	2e	-
STEC214	Pig	Beijing	2012	O100	H30	-	2e	-

STEC215	Pig	Beijing	2012	O20	H30	-	2e	-
STEC216	Pig	Beijing	2012	O20	H30	-	2e	-
STEC217	Pig	Beijing	2012	O20	H30	-	2e	-
STEC218	Pig	Beijing	2012	O20	H30	-	2e	-
STEC219	Pig	Beijing	2012	O114	H30	-	2e	-
STEC220	Pig	Beijing	2012	O7	H42	-	2e	-
STEC221	Pig	Beijing	2012	O172	H30	-	2e	-
STEC222	Pig	Beijing	2012	NT	H30	-	2e	-
STEC223	Pig	Beijing	2012	NT	H30	-	2e	-
STEC224	Pig	Beijing	2012	NT	H30	-	2e	-
STEC225	Pig	Beijing	2012	NT	H30	-	2e	-
STEC226	Pig	Beijing	2012	NT	H30	-	2e	-
STEC227	Pig	Beijing	2012	NT	H30	-	2e	-
STEC228	Pig	Beijing	2012	NT	H30	-	2e	-
STEC229	Pig	Beijing	2012	NT	H20	-	2e	-
STEC230	Pig	Beijing	2012	NT	H20	-	2e	-
STEC231	Pig	Beijing	2012	NT	NT	-	2e	-
STEC232	Pig	Beijing	2012	NT	H20	-	2e	-
STEC233	Pig	Beijing	2012	O65	H30	-	2e	-
STEC234	Pig	Beijing	2012	O142	H27	-	2e	-
STEC235	Pig	Beijing	2012	O86	H11	-	2e	-
STEC236	Pig	Beijing	2012	NT	H30	-	2e	-
STEC237	Pig	Beijing	2012	O20	H30	-	2e	-
STEC238	Pig	Beijing	2012	O9	H30	-	2e	-
STEC239	Pig	Beijing	2012	NT	H30	-	2e	-
STEC240	Pig	Beijing	2012	O143	H38	-	2e	-
STEC241	Pig	Beijing	2012	O143	H38	-	2e	-
STEC242	Pig	Beijing	2012	NT	H17	-	2e	-
STEC243	Pig	Beijing	2012	NT	H17	-	2e	-
STEC244	Pig	Beijing	2012	O5(O70)	H30	-	2e	-
STEC245	Pig	Beijing	2012	O5(O70)	H30	-	2e	-
STEC246	Pig	Beijing	2012	O65	H30	-	2e	-
STEC247	Pig	Beijing	2012	O20	H30	-	2e	-
STEC248	Pig	Beijing	2012	NT	H30	-	2e	-
STEC249	Pig	Beijing	2012	NT	H30	-	2e	-
STEC250	Pig	Beijing	2012	O20	H30	-	2e	-
STEC251	Pig	Beijing	2012	NT	H30	-	2e	-
STEC252	Pig	Beijing	2012	O9	H30	-	2e	-
STEC253	Pig	Beijing	2012	O100	H30	-	2e	-
STEC254	Pig	Beijing	2012	O5(O70)	H30	-	2e	-
STEC255	Pig	Beijing	2012	O86	H11	-	2e	-
STEC256	Pig	Beijing	2012	O9	H30	-	2e	-
STEC257	Pig	Beijing	2012	O9	H30	-	2e	-
STEC258	Pig	Beijing	2012	O20	H30	-	2e	-
STEC259	Pig	Beijing	2012	O71	H30	-	2e	-
STEC260	Pig	Beijing	2012	O116	H30	-	2e	-
STEC261	Pig	Beijing	2012	O65	H30	-	2e	-
STEC262	Pig	Beijing	2012	O65	H30	-	2e	-
STEC263	Pig	Beijing	2012	O65	H30	-	2e	-
STEC264	Pig	Beijing	2012	NT	H30	-	2e	-
STEC265	Pig	Beijing	2012	O100	H30	-	2e	-
STEC266	Pig	Beijing	2012	O143	H38	-	2e	-
STEC267	Pig	Beijing	2012	NT	H19	-	2e	-
STEC268	Pig	Harbin	2012	O100	H30	-	2	-

STEC269	Pig	Harbin	2012	O173	H21	-	2	-
STEC270	Pig	Harbin	2012	O2 (O50)	H30	-	2	-
STEC271	Pig	Harbin	2012	O2 (O50)	H30	-	2	-
STEC272	Pig	Harbin	2012	O2 (O50)	H30	-	2	-
STEC273	Pig	Harbin	2012	O2 (O50)	H32	-	2	-
STEC274	Pig	Harbin	2012	O2 (O50)	H32	-	2	-
STEC275	Pig	Harbin	2012	O168	H38	-	2	-
STEC276	Beef cattle	Harbin	2012	NT	H30	-	2g	-
STEC277	Beef cattle	Harbin	2012	O71	H30	-	2	-
STEC278	Beef cattle	Harbin	2012	NT	H30	-	2g	-
STEC280	Beef cattle	Harbin	2012	NT	H30	-	2g	-
STEC281	Beef cattle	Harbin	2012	O22	H8	1a	2d	-
STEC282	Cow	Heilongjiang	2012	O181	H4	-	2d	-
STEC283	Cow	Heilongjiang	2012	NT	H9	-	2g	-
STEC284	Cow	Heilongjiang	2012	NT	H9	-	2g	-
STEC285	Cow	Heilongjiang	2012	O81	H31	-	2d	-
STEC286	Cow	Heilongjiang	2012	O81	H31	-	2d	-
STEC287	Beef cattle	Heilongjiang	2012	O99	H10	-	2	-
STEC288	Cow	Heilongjiang	2012	O81	H31	-	2d	-
STEC289	*Marmota himalayana*	Qinghai	2012	O112ab	H8	1	-	-
STEC291	*Marmota himalayana*	Qinghai	2012	O5	NT	1	-	-
STEC292	*Marmota himalayana*	Qinghai	2012	O5	NT	1	-	-
STEC293	*Marmota himalayana*	Qinghai	2013	O53	H18	-	2h	-
STEC294	*Marmota himalayana*	Qinghai	2013	O53	H18	-	2h	-
STEC295	*Marmota himalayana*	Qinghai	2013	O53	H18	-	2h	-
STEC296	*Marmota himalayana*	Qinghai	2013	O71	H8	-	2a	-
STEC298	Plateau Pika	Qinghai	2013	O74	H8	-	2d	-
STEC299	*Marmota himalayana*	Qinghai	2013	O53	H18	-	2h	-
STEC300	Raw beef	Sichuan	2013	O96	H19	-	2a	-
STEC301	pork	Sichuan	2013	O141	H29	-	2e	-
STEC302	Raw beef	Sichuan	2013	O22	H16	-	2b	-
STEC304	Raw beef	Beijing	2013	O40	H8	-	2a	-
STEC305	Raw beef	Beijing	2013	O40	H8	-	2a	-
STEC306	Food	Beijing	2013	O8	H9	-	2e	-
STEC307	Food	Beijing	2013	O8	H9	-	2e	-
STEC308	Food	Beijing	2013	O8	H9	-	2e	-
STEC309	Diarrheal patient	Sichuan	2013	O48	H21	-	2e	-
STEC310	Diarrheal patient	Shanghai	2013	O5	NT	1	-	+
STEC311	Diarrheal patient	Shanghai	2013	O68	H10	1	-	-
STEC312	Diarrheal patient	Shanghai	2013	O26	H11	1	-	+
STEC313	Raw beef	Beijing	2013	O100	H19	-	2e	-
STEC314	Raw pork	Beijing	2013	O98	H10	-	2e	-
STEC315	Raw pork	Beijing	2013	O98	H30	-	2e	-
STEC316	Raw beef	Beijing	2014	O100	H19	-	2e	-
STEC317	Raw mutton	Beijing	2014	NT	H21	1c	-	-
STEC318	Raw pork	Beijing	2014	O121	H10	-	2e	-
STEC319	Raw mutton	Beijing	2014	O176	H4	1c	2b	-
STEC320	Raw mutton	Beijing	2014	O5	H19	1c	2b	-
STEC321	Raw mutton	Beijing	2014	O128	H2	1c	2b	-
STEC322	Raw chicken meat	Beijing	2014	O176	H4	1c	-	-
STEC323	Raw duck meat	Beijing	2014	O176	H4	1c	-	-
STEC325	unknown	unknown	unknown	O174	H8	1c	2b	-
STEC326	unknown	unknown	unknown	O8	NT	1d	-	-
STEC327	unknown	unknown	unknown	O118	H12	-	2b	-

STEC328	unknown	unknown	unknown	O139	H1	-	2e	-
STEC329	unknown	unknown	unknown	O128ac	H2	-	2f	+
STEC330	unknown	unknown	unknown	O2	H25	-	2g	-
STEC332	unknown	unknown	unknown	O166	H15	-	2d	-
STEC333	unknown	unknown	unknown	O113	H4	1c	2b	-
STEC334	unknown	unknown	unknown	O121	H19	-	2a	+
STEC335	unknown	unknown	unknown	O128ab	NT	1c	-	-
STEC336	unknown	unknown	unknown	O41	H26	1d	-	-
STEC337	unknown	unknown	unknown	O26	H11	-	2a	+
STEC338	unknown	unknown	unknown	O111	NT	1a	-	+
STEC340	unknown	unknown	unknown	O146	H21	-	2d	-
STEC341	unknown	unknown	unknown	O103	H2	1a	-	+
STEC343	unknown	unknown	unknown	O166	H15	-	2d	-
STEC344	Raw mutton	Beijing	2014	O8	H30	-	2e	-
STEC345	Raw pork	Beijing	2014	O57	H21	-	2e	-
STEC346	Raw pork	Beijing	2014	O8	H19	-	2e	-
STEC347	Raw beef	Beijing	2014	O110	H16	1c	-	-
STEC348	Raw beef	Beijing	2014	O103	H25	1a	-	+
STEC349	Raw beef	Beijing	2014	O98	H30	-	2e	-
STEC350	Raw beef	Beijing	2014	O103	H25	1a	-	+
STEC351	Raw beef	Beijing	2014	O98	H30	-	2e	-
STEC352	Raw mutton	Beijing	2014	O120	H16	1c	-	-
STEC353	Raw beef	Beijing	2014	O128	H2	1c	2b	-
STEC354	Raw beef	Beijing	2014	O128	H2	1c	2b	-
STEC355	Raw beef	Beijing	2014	O128	H2	1c	2b	-
STEC356	Raw mutton	Beijing	2014	O161	H19	1c	-	-
STEC357	Raw mutton	Beijing	2014	O176	H4	1c	-	-
STEC358	Raw mutton	Beijing	2014	O104	H7	1c	-	-
STEC359	Raw mutton	Beijing	2014	O128	H2	1c	2b	-
STEC360	Raw mutton	Beijing	2014	O176	H4	1c	-	-
STEC361	Raw mutton	Beijing	2014	O21	H25	1a	-	-
STEC362	Raw mutton	Beijing	2014	O91	H14	1a	2b	-
STEC363	Raw mutton	Beijing	2014	O104	H7	1c	-	-
STEC364	Raw mutton	Beijing	2014	O128	H2	1c	2b	-
STEC365	Raw beef	Beijing	2014	O12	NT	-	2c	+
STEC366	Raw beef	Beijing	2014	O128	H2	1c	-	-
STEC367	Raw mutton	Beijing	2014	O100	H19	-	2e	-
STEC368	Raw mutton	Beijing	2014	O104	H7	1c	-	-
STEC369	Raw mutton	Beijing	2014	O22	H16	-	2d	-
STEC370	Raw mutton	Sichuan	2014	O113	H7	1c	-	-
STEC371	Raw beef	Sichuan	2014	O84	H20	1d	-	-
STEC372	Raw beef	Sichuan	2014	O76	H21	1a	-	-
STEC373	Raw mutton	Sichuan	2014	O21	H25	1a	-	-
STEC374	Raw beef	Beijing	2014	O8	H30	-	2a	-
STEC375	Raw beef	Beijing	2014	O20	H21	-	2e	-
STEC376	Raw mutton	Beijing	2014	O128	H2	1c	2b	-
STEC377	Raw mutton	Beijing	2014	O5	H9	1a	-	+
STEC378	Raw beef	Beijing	2014	O116	H21	1a	2a	-
STEC379	Raw pork	Beijing	2014	O8	H19	-	2e	-
STEC380	Pig	Beijing	2014	O141	H29	-	2e	-
STEC381	Raw mutton	Beijing	2014	O120	H30	-	2e	-
STEC382	Pig	Beijing	2014	O91	H4	-	2e	-
STEC383	Pig	Beijing	2014	O172	H30	-	2e	-
STEC384	Raw pork	Beijing	2014	O8	H19	-	2e	-

STEC385	Raw pork	Beijing	2014	O91	H4	-	2e	-
STEC386	Raw pork	Beijing	2014	O91	H4	-	2e	-
STEC387	Raw pork	Beijing	2014	O91	H4	-	2e	-
STEC388	Pig	Guizhou	2012	O159	H16	-	2e	-
STEC389	Pig	Guizhou	2012	O100	H20	-	2e	-
STEC390	Pig	Guizhou	2012	NT	H7	-	2e	-
STEC391	Pig	Guizhou	2012	O87	H10	-	2e	-
STEC392	Pig	Guizhou	2012	O100	H20	-	2e	-
STEC393	Pig	Guizhou	2012	NT	H7	-	2e	-
STEC394	unknown	Heilongjiang	2014	O26	H11 -	-	2	+
STEC395	unknown	Guangxi	2014	O55	H7 -	1	-	+
STEC397	Diarrheal patient	Henan	2013	O130	H8	1c	-	-
STEC398	Diarrheal patient	Henan	2013	O130	H8	1c	-	-
STEC399	Diarrheal patient	Henan	2013	O130	H8	1c	-	-
STEC400	Diarrheal patient	Henan	2013	O130	H8	1c	-	-
STEC401	Diarrheal patient	Henan	2013	O130	H8	1c	-	-
STEC402	Diarrheal patient	Henan	2013	O117	H8	1c	-	-
STEC403	Diarrheal patient	Henan	2013	O117	H8	1c	-	-
STEC404	Diarrheal patient	Henan	2013	O130	H8	1c	-	-
STEC405	Diarrheal patient	Henan	2013	O117	H8	1c	-	-
STEC406	Diarrheal patient	Shanghai	2012	O26	H11	1a	-	+
STEC407	Diarrheal patient	Shanghai	2013	O5	NT	1a	-	+
STEC408	Diarrheal patient	Shanghai	2013	O21	H25	1a	-	-
STEC409	Diarrheal patient	Shanghai	2013	NT	H30	-	2e	-
STEC410	Diarrheal patient	Shanghai	2013	O149	H10	1c	-	-
STEC411	Diarrheal patient	Shanghai	2013	O26	H11	1a	-	+
STEC412	Diarrheal patient	Shanghai	2013	O112ab	H8	1a	-	-
STEC413	Diarrheal patient	Shanghai	2014	O20	H30	-	2g	-
STEC414	Diarrheal patient	Shanghai	2014	O104	H7	1c	-	-
STEC415	Diarrheal patient	Shanghai	2014	O84	H2	1a	-	+
STEC416	Diarrheal patient	Shanghai	2014	O26	H11	1a	-	+
STEC417	unknown	unknown	2014	O104	H4	-	2a	-
STEC419	unknown	unknown	2014	O111	H8	1a	-	+
STEC420	unknown	unknown	2014	O146	H28	-	2b	-
STEC422	unknown	unknown	2014	NT	H7	1c	2b	-
STEC424	unknown	unknown	2014	O97	H26	1d	-	-
STEC425	unknown	unknown	2014	O26	H11	1a	-	+
STEC426	unknown	unknown	2014	O121	H19	-	2a	+
STEC427	unknown	unknown	2014	O166	H15	-	2d	-
STEC428	unknown	unknown	2014	O68	H14	-	2b	-
STEC429	unknown	unknown	2014	O55	H7	1a	-	+
STEC430	unknown	unknown	2014	O68	H2	1a	-	+
STEC432	unknown	unknown	2014	O111	H8	1a	-	+
STEC433	unknown	unknown	2014	O26	H11	1a	-	+
STEC434	Healthy carrier	Guangdong	2014	O43	H2	-	2d	-
STEC435	Healthy carrier	Qinghai	2013	O91	H14	1a	2b	-
STEC437	Diarrheal patient	Guangdong	2010	O111	H8	1a	-	-
STEC438	Healthy carrier	Guangdong	2014	NT	H21	1c	-	-
STEC439	Plateau Pika	Qinghai	2015	O169	H8	-	2b	-
STEC440	Plateau Pika	Qinghai	2015	O74	H8	-	2a+2d	-
STEC441	Plateau Pika	Qinghai	2015	O49	H21	-	2b	-
STEC442	Plateau Pika	Qinghai	2015	O2	H45	1a	-	-
STEC443	Plateau Pika	Qinghai	2015	O119	H19	1a	-	-
STEC444	Plateau Pika	Qinghai	2015	O49	H21	-	2b	-

STEC445	Plateau Pika	Qinghai	2015	O81	H21	-	2a	-
STEC446	Plateau Pika	Qinghai	2015	O96	H8	-	2d	-
STEC447	Plateau Pika	Qinghai	2015	O2	H45	1a	-	-
STEC448	Plateau Pika	Qinghai	2015	O120	H9	1a	2d	-
STEC449	Plateau Pika	Qinghai	2015	O163	H19	-	2b	-
STEC450	Plateau Pika	Qinghai	2015	O159	H21	1a	2d	-
STEC451	Plateau Pika	Qinghai	2012	O8	H16	-	2b	-
STEC452	Plateau Pika	Qinghai	2012	O8	H2	-	2d	-
STEC453	Plateau Pika	Qinghai	2012	O8	H2	-	2d	-
STEC454	Plateau Pika	Qinghai	2012	O8	H2	-	2d	-
STEC455	Plateau Pika	Qinghai	2012	O82	H19	1a	-	-
STEC456	Plateau Pika	Qinghai	2012	O2	H45	1a	-	-
STEC457	Plateau Pika	Qinghai	2012	O170	H8	-	2d	-
STEC458	Plateau Pika	Qinghai	2012	O74	H8	1a	2d	-
STEC459	Plateau Pika	Qinghai	2012	O8	H2	-	2d	-
STEC461	Beef cattle	Shandong	2015	O177	NT	-	2c	+
STEC463	Pig	Shandong	2015	O77	H30	-	2e	-
STEC464	Pig	Shandong	2015	O77	H30	-	2e	-
STEC465	Pig	Shandong	2015	O86	H32	-	2e	-
STEC466	Pig	Shandong	2015	O44	H30	-	2e	-
STEC467	Pig	Shandong	2015	O110 (O113)	H30	-	2e	-
STEC468	Pig	Shandong	2015	O20 O60	H30	-	2e	-
STEC469	Pig	Shandong	2015	O10	H30	-	2e	-
STEC470	Pig	Shandong	2015	O110	NT	-	2e	-
STEC471	Pig	Shandong	2015	O114	H27	-	2e	-
STEC472	Pig	Shandong	2015	O20	H27	-	2e	-
STEC473	Pig	Shandong	2015	O65	H9	-	2e	-
STEC474	Pig	Shandong	2015	O65	H9	-	2e	-
STEC475	Pig	Shandong	2015	O8	NT	-	2e	-
STEC476	Tibetan antelope	Qinghai	2014	O102	H8	1a	-	-
STEC477	Tibetan antelope	Qinghai	2014	O50	H14	1a	-	-
STEC484	Tibetan antelope	Qinghai	2014	O105	H8	-	2	-
STEC485	Tibetan antelope	Qinghai	2014	O74	H8	-	2	-
STEC486	Tibetan antelope	Qinghai	2014	O74	H8	-	2	-
STEC506	Diarrheal patient	Shandong	2014	O3	H4	1	-	-
STEC507	Diarrheal patient	Shandong	2016	O3	H28	1	-	-
STEC508	Diarrheal patient	Shanghai	2016	O26	H11	1	-	+
STEC509	Healthy carrier	Guangdong	2016	O8	H19	-	2	-
STEC510	Beef cattle	Sichuan	2017	O81	H31	1a	2a	-
STEC511	Beef cattle	Sichuan	2017	NT	H8	-	2a	-
STEC512	Beef cattle	Sichuan	2017	O84	H2	1a	-	+
STEC513	Beef cattle	Sichuan	2017	NT	H8	-	2a	-
STEC514	Beef cattle	Sichuan	2017	NT	H8	-	2a	-
STEC515	Beef cattle	Sichuan	2017	O81	H31	1a	2a	-
STEC516	Beef cattle	Sichuan	2017	NT	H8	-	2a	-
STEC517	Beef cattle	Sichuan	2017	O81	H31	1a	2d	-
STEC518	Beef cattle	Sichuan	2017	O65	H19	-	2d	-
STEC519	Beef cattle	Sichuan	2017	O84	H2	1a	-	+
STEC520	Beef cattle	Sichuan	2017	O5(070)	H31	1a	2c	-
STEC521	Beef cattle	Sichuan	2017	O84	H2	1a	-	+
STEC522	Beef cattle	Sichuan	2017	O177	NT	-	2c	+
STEC523	Beef cattle	Sichuan	2017	O84	H2	1a	-	+
STEC524	Beef cattle	Sichuan	2017	NT	H8	-	2a	-
STEC525	Beef cattle	Sichuan	2017	O81	H31	1a	2d	-

STEC526	Beef cattle	Sichuan	2017	O5(070)	H31	1a	2c	-
STEC527	Beef cattle	Sichuan	2017	O84	H2	1a	-	+
STEC528	Beef cattle	Sichuan	2017	NT	H8	-	2a	-
STEC529	Beef cattle	Sichuan	2017	O8	H19	1a	2a	-
STEC530	Beef cattle	Sichuan	2017	O65	H19	-	2c	-
STEC531	Beef cattle	Sichuan	2017	O5(070)	H31	1a	2c	-
STEC532	Beef cattle	Sichuan	2017	O84	H2	1a	-	+
STEC533	Beef cattle	Sichuan	2017	NT	H8	-	2a	-
STEC534	Beef cattle	Sichuan	2017	O65	H19	-	2c	-
STEC535	Beef cattle	Sichuan	2017	O74	H8	1a	2d	-
STEC536	Beef cattle	Sichuan	2017	O84	H2	1a	-	+
STEC537	Beef cattle	Sichuan	2017	O84	H2	1a	-	+
STEC538	Beef cattle	Sichuan	2017	NT	H8	-	2a	-
STEC539	Beef cattle	Sichuan	2017	O81	H31	1a	2d	-
STEC540	Beef cattle	Sichuan	2017	O84	H2	1a	-	+
STEC541	Beef cattle	Sichuan	2017	NT	H8	-	2a	-
STEC542	Beef cattle	Sichuan	2017	O84	H2	1a	-	+
STEC543	Beef cattle	Sichuan	2017	NT	H8	-	2a	-
STEC544	Beef cattle	Sichuan	2017	O84	H2	1a	-	+
STEC545	Beef cattle	Sichuan	2017	NT	H8	-	2a	-
STEC546	Beef cattle	Sichuan	2017	O84	H2	1a	-	+
STEC547	Beef cattle	Sichuan	2017	O81	H31	1a	2c	-
STEC548	Beef cattle	Sichuan	2017	O145	H12	1a	-	-
STEC549	Beef cattle	Sichuan	2017	O81	H31	1a	2d	-
STEC550	Beef cattle	Sichuan	2017	O84	H2	1a	-	+
STEC551	Beef cattle	Sichuan	2017	O84	H2	1a	-	+
STEC552	Beef cattle	Sichuan	2017	NT	H8	-	2a	-
STEC553	Beef cattle	Sichuan	2017	O5(070)	H31	1a	2c	-
STEC554	Beef cattle	Sichuan	2017	O84	H2	1a	-	+
STEC555	Beef cattle	Sichuan	2017	O177	NT	-	2c	+
STEC556	Beef cattle	Sichuan	2017	O5(070)	H31	1a	2c	-
STEC557	Beef cattle	Sichuan	2017	O177	NT	-	2c	+
STEC558	Beef cattle	Sichuan	2017	O81	H31	1a	2d	-
STEC559	Beef cattle	Sichuan	2017	O76	H21	-	2d	-
STEC560	Beef cattle	Sichuan	2017	O8	H19	1a	2a	-
STEC561	Beef cattle	Sichuan	2017	O96	H29	-	2d	-
STEC562	Beef cattle	Sichuan	2017	O81	H31	1a	2d	-
STEC563	Beef cattle	Sichuan	2017	O8	H19	1a	2a	-
STEC564	Beef cattle	Sichuan	2017	O81	H31	1a	2c	-
STEC565	Beef cattle	Sichuan	2017	O15	H29	-	2d	-
STEC566	Beef cattle	Sichuan	2017	O84	H2	1a	-	+
STEC567	Beef cattle	Sichuan	2017	O81	H31	1a	2d	-
STEC568	Beef cattle	Sichuan	2017	O5(070)	H31	1a	2c	-
STEC569	Beef cattle	Sichuan	2017	O65	H19	-	2c	-
STEC570	Beef cattle	Sichuan	2017	O177	NT	-	2c	+
STEC571	Beef cattle	Sichuan	2017	NT	H8	-	2a	-
STEC572	Beef cattle	Sichuan	2017	O22	H16	-	2d	-
STEC573	Beef cattle	Sichuan	2017	O81	H31	1a	2c	-
STEC574	Beef cattle	Sichuan	2017	O177	NT	-	2c	+
STEC575	Beef cattle	Sichuan	2017	O177	NT	-	2c	+
STEC576	Beef cattle	Sichuan	2017	O81	H31	1a	2a	-
STEC577	Beef cattle	Sichuan	2017	O165	H8	-	2a	-
STEC578	Beef cattle	Sichuan	2017	O5(070)	H31	1a	2c	-
STEC579	Beef cattle	Sichuan	2017	O84	H2	1a	-	+

STEC580	Beef cattle	Sichuan	2017	O5(070)	H31	1a	2c	-
STEC581	Beef cattle	Sichuan	2017	O81	H31	1a	2d	-
STEC582	Beef cattle	Sichuan	2017	O96	H29	-	2d	-
STEC583	Beef cattle	Sichuan	2017	NT	H8	-	2a	-
STEC584	Beef cattle	Sichuan	2017	O81	H31	1a	2d	-
STEC585	Beef cattle	Sichuan	2017	NT	H8	-	2a	-
STEC586	Beef cattle	Sichuan	2017	O5(070)	H31	1a	2d	-
STEC587	Beef cattle	Sichuan	2017	NT	H8	-	2a	-
STEC588	Beef cattle	Sichuan	2017	O81	H31	1a	2a	-
STEC589	Beef cattle	Sichuan	2017	O166	H29	-	2d	-
STEC590	Beef cattle	Sichuan	2017	O5(070)	H31	1a	2c	-
STEC591	Beef cattle	Sichuan	2017	O84	H2	1a	-	+
STEC592	Beef cattle	Sichuan	2017	NT	H8	-	2a	-
STEC593	Beef cattle	Sichuan	2017	O5(070)	H31	1a	2c	-
STEC594	Beef cattle	Sichuan	2017	NT	H8	-	2a	-
STEC595	Beef cattle	Sichuan	2017	NT	H8	-	2a	-
STEC596	Beef cattle	Sichuan	2017	O96	H29	-	2d	-
STEC597	Beef cattle	Sichuan	2017	O5(070)	H31	1a	2c	-
STEC598	Beef cattle	Sichuan	2017	NT	H8	-	2a	-
STEC599	Beef cattle	Sichuan	2017	O5(070)	H31	1a	2c	-
STEC600	Beef cattle	Sichuan	2017	O84	H2	1a	-	+
STEC601	Beef cattle	Sichuan	2017	O84	H2	1a	-	+
STEC602	Beef cattle	Sichuan	2017	O84	H2	1a	-	+
STEC603	Beef cattle	Sichuan	2017	O81	H31	1a	2d	-
STEC604	Beef cattle	Sichuan	2017	O84	H2	1a	-	+
STEC605	Beef cattle	Sichuan	2017	NT	H8	-	2a	-
STEC606	Beef cattle	Sichuan	2017	O81	H31	1a	2d	-
STEC607	Beef cattle	Sichuan	2017	O84	H2	1a	-	+
STEC608	Beef cattle	Sichuan	2017	NT	H8	-	2a	-
STEC609	Beef cattle	Sichuan	2017	O5(070)	H31	1a	2c	-
STEC610	Beef cattle	Sichuan	2017	O8	H19	1a	2a	-
STEC611	Beef cattle	Sichuan	2017	O177	NT	-	2c	+
STEC612	Beef cattle	Sichuan	2017	O81	H31	1a	2d	-
STEC613	Beef cattle	Sichuan	2017	O84	H2	1a	-	+
STEC614	Beef cattle	Sichuan	2017	O5(070)	H31	1a	2c	-
STEC615	Beef cattle	Sichuan	2017	O166	H29	-	2d	-
STEC616	Beef cattle	Sichuan	2017	O84	H2	1a	-	+
STEC617	Beef cattle	Sichuan	2017	O81	H31	1a	2c	-
STEC618	Beef cattle	Sichuan	2017	O81	H31	1a	2d	-
STEC619	Beef cattle	Sichuan	2017	O22	H16	-	2d	-
STEC620	Beef cattle	Sichuan	2017	O84	H2	1a	-	+
STEC621	Beef cattle	Sichuan	2017	O81	H31	1a	2d	-
STEC622	Beef cattle	Sichuan	2017	O5(070)	H31	1a	2c	-
STEC623	Beef cattle	Sichuan	2017	O84	H2	1a	-	+
STEC624	Beef cattle	Sichuan	2017	O81	H31	1a	2d	-
STEC625	Beef cattle	Sichuan	2017	O116	H10	1a	2a	-
STEC626	Beef cattle	Sichuan	2017	O81	H31	1a	2a	-
STEC627	Beef cattle	Sichuan	2017	O8	H19	1a	2a	-
STEC628	Beef cattle	Sichuan	2017	O84	H2	1a	-	+
STEC629	Beef cattle	Sichuan	2017	O84	H2	1a	-	+
STEC630	Beef cattle	Sichuan	2017	O81	H31	-	2a	-
STEC631	Beef cattle	Sichuan	2017	O5(070)	H31	1a	2c	-
STEC632	Beef cattle	Sichuan	2017	O81	H29	-	2d	-
STEC633	Beef cattle	Sichuan	2017	O84	H2	1a	-	-

STEC634	Beef cattle	Sichuan	2017	O81	H31	1a	2d	-
STEC635	Beef cattle	Sichuan	2017	O8	H19	1a	2a	-
STEC636	Goat	Sichuan	2017	NT	H21	1c	-	-
STEC637	Goat	Sichuan	2017	NT	H21	1c	-	-
STEC638	Goat	Sichuan	2017	NT	H21	1c	-	-
STEC639	Goat	Sichuan	2017	O21	H25	1a	-	-
STEC640	Goat	Sichuan	2017	O22	H16	-	2b	-
STEC641	Goat	Sichuan	2017	O22	H16	-	2b	-
STEC642	Goat	Sichuan	2017	O22	H16	-	2b	-
STEC643	Goat	Sichuan	2017	O22	H16	-	2b	-
STEC644	Goat	Sichuan	2017	O22	H16	-	2b	-
STEC645	Goat	Sichuan	2017	O22	H16	-	2b	-
STEC646	Goat	Sichuan	2017	O22	H16	-	2b	-
STEC647	Goat	Sichuan	2017	O22	H16	-	2b	-
STEC648	Goat	Sichuan	2017	NT	H8	1c	-	-
STEC649	Goat	Sichuan	2017	O22	H16	-	2b	-
STEC650	Goat	Sichuan	2017	O21	H25	1a	-	-
STEC651	Goat	Sichuan	2017	O22	H16	-	2b	-
STEC652	Goat	Sichuan	2017	O22	H16	-	2b	-
STEC653	Goat	Sichuan	2017	O21	H25	1a	-	-
STEC654	Goat	Sichuan	2017	NT	H25	1c	-	-
STEC655	Goat	Sichuan	2017	O22	H16	-	2b	-
STEC656	Goat	Sichuan	2017	NT	H21	1c	-	-
STEC657	Goat	Sichuan	2017	O22	H16	-	2b	-
STEC658	Goat	Sichuan	2017	O21	H25	1a	-	-
STEC659	Goat	Sichuan	2017	O21	H25	1a	-	-
STEC660	Goat	Sichuan	2017	O21	H25	1a	-	-
STEC661	Goat	Sichuan	2017	O21	H25	1a	-	-
STEC662	Goat	Sichuan	2017	O21	H25	1a	-	-
STEC663	Goat	Sichuan	2017	O21	H25	1a	-	-
STEC664	Goat	Sichuan	2017	O71	H19	1c	-	-
STEC665	Goat	Sichuan	2017	O21	H25	1a	-	-
STEC666	Goat	Sichuan	2017	O21	H25	1a	-	-
STEC667	Goat	Sichuan	2017	O21	H25	1a	-	-
STEC668	Goat	Sichuan	2017	O21	H25	1a	-	-
STEC669	Goat	Sichuan	2017	O21	H25	1a	-	-
STEC670	Goat	Sichuan	2017	O21	H25	1a	-	-
STEC671	Goat	Sichuan	2017	O21	H25	1a	-	-
STEC672	Goat	Sichuan	2017	O21	H25	1a	-	-
STEC673	Goat	Sichuan	2017	O21	H25	1a	-	-
STEC674	Goat	Sichuan	2017	O21	H25	1a	-	-
STEC675	Goat	Sichuan	2017	O21	H25	1a	-	-
STEC676	Goat	Sichuan	2017	O21	H25	1a	-	-
STEC677	Goat	Sichuan	2017	O21	H25	1a	-	-
STEC678	Goat	Sichuan	2017	O21	H25	1a	-	-
STEC679	Goat	Sichuan	2017	O21	H25	1a	-	-
STEC680	Goat	Sichuan	2017	O21	H25	1a	-	-
STEC681	Goat	Sichuan	2017	O21	H25	1a	-	-
STEC682	Goat	Sichuan	2017	O21	H25	1a	-	-
STEC683	Goat	Sichuan	2017	O21	H25	1a	-	-
STEC684	Goat	Sichuan	2017	O22	H16	-	2b	-
STEC685	Goat	Sichuan	2017	O22	H16	-	2b	-
STEC686	Goat	Sichuan	2017	O150	H8	1c	-	-
STEC687	Goat	Sichuan	2017	O116	H19	1c	-	-

STEC688	Goat	Sichuan	2017	O22	H16	-	2b	-
STEC689	Beef cattle	Shandong	2017	O44	NT	1a	-	+
STEC690	Beef cattle	Shandong	2017	O116	NT	-	2c	+
STEC691	Beef cattle	Shandong	2017	O5	NT	-	2c	+
STEC692	Goat	Shandong	2017	O118	H21	1	-	-
STEC693	Goat	Shandong	2017	O50	H2	1	-	-
STEC694	Goat	Shandong	2017	O53	H2	1	-	-
STEC695	Goat	Shandong	2017	O50	H25	1	-	-
STEC696	Goat	Shandong	2017	O74	NT	1	2	-
STEC697	Goat	Shandong	2017	O74	NT	1	-	-
STEC698	Beef cattle	Shandong	2017	O55	H10	1	-	-
STEC699	Beef cattle	Shandong	2017	O28	H10	1	-	-
STEC700	Goat	Shandong	2017	O115	H25	1	-	-
STEC701	Goat	Shandong	2017	OUT	H4	1	-	-
STEC702	Goat	Shandong	2017	O44	H21	-	2	-
STEC703	Beef cattle	Shandong	2017	O75	H29	-	2	-
STEC704	Goat	Shandong	2017	O115	H28	-	2	-
STEC705	Goat	Shandong	2017	O110	H28	-	2	-
STEC706	Beef cattle	Shandong	2017	O19	NT	-	2	-
STEC707	Beef cattle	Shandong	2017	O71	H8	-	2	-
STEC708	Beef cattle	Shandong	2017	O2	NT	-	2	-
STEC709	Goat	Shandong	2017	O116	H28	-	2	-
STEC710	Goat	Shandong	2017	O74	H28	-	2	-
STEC711	Goat	Shandong	2017	O74	H8	-	2	-
STEC712	Goat	Shandong	2017	O65	H51	-	2	-
STEC713	Goat	Shandong	2017	O50	H28	-	2	-
STEC714	Goat	Shandong	2017	O50	H28	-	2	-
STEC715	Goat	Shandong	2017	O50	H28	-	2	-
STEC716	Goat	Shandong	2017	O19	H28	-	2	-
STEC717	Goat	Shandong	2017	OUT	H31	-	2	-
STEC718	Goat	Shandong	2017	O116	H30	-	2	-
STEC719	Goat	Shandong	2017	O65	H28	-	2	-
STEC720	Goat	Shandong	2017	O55	H8	-	2	-
STEC721	Goat	Shandong	2017	O19	H28	-	2	-
STEC722	Goat	Shandong	2017	O65	H28	-	2	-
STEC723	Goat	Shandong	2017	O28	H25	-	2	-
STEC724	Goat	Shandong	2017	O19	H8	-	2	-
STEC725	Goat	Shandong	2017	O23	H25	-	2	-
STEC726	Goat	Shandong	2017	O55	NT	-	2	-
STEC727	Goat	Shandong	2017	O23	H4	1	2	-
STEC728	Goat	Shandong	2017	O23	H4	1	2	-
STEC729	Goat	Shandong	2017	O74	H4	1	2	-
STEC730	Goat	Shandong	2017	O23	H4	1	2	-
STEC731	Goat	Shandong	2017	O74	H4	1	2	-
STEC732	Goat	Shandong	2017	O23	H4	1	2	-
STEC733	Goat	Shandong	2017	O23	H7	1	2	-
STEC734	Beef cattle	Shandong	2017	O38	H31	1	2	-
STEC735	Goat	Shandong	2017	O23	H4	1	2	-
STEC736	Goat	Shandong	2017	O23	H4	1	2	-
STEC737	Goat	Shandong	2017	O23	H4	1	2	-
STEC738	Goat	Shandong	2017	O23	H4	1	2	-
STEC739	Beef cattle	Shandong	2017	O23	H10	1	2	-
STEC740	Goat	Shandong	2017	O50	H4	1	2	-
STEC741	Beef cattle	Shandong	2017	O30	H38	1	2	-

STEC742	Goat	Shandong	2017	O114	H19	1	2	-
STEC743	Goat	Shandong	2017	O7	H19	1	2	-
STEC744	Goat	Shandong	2017	O23	H4	1	2	-
STEC745	Goat	Shandong	2017	O23	H4	1	2	-
STEC746	Goat	Shandong	2017	O23	H4	1	2	-
STEC747	Goat	Shandong	2017	O3	H4	1	2	-
STEC748	Goat	Shandong	2017	O3	H4	1	2	-
STEC749	Goat	Shandong	2017	O23	H4	1	2	-
STEC750	Goat	Shandong	2017	O19	H16	1	2	-
STEC751	Goat	Shandong	2017	O74	H4	1	2	-
STEC752	Goat	Shandong	2017	O3	H4	1	2	-
STEC753	Goat	Shandong	2017	O23	H4	1	2	-
STEC754	Goat	Shandong	2017	O23	H4	1	2	-
STEC755	Goat	Shandong	2017	O5	H19	1	2	-
STEC756	Goat	Shandong	2017	O62	H8	1	2	-
STEC757	Goat	Shandong	2017	O39	H2	1	2	-
STEC758	Goat	Shandong	2017	O115	H2	1	2	-
STEC759	Goat	Shandong	2017	O74	H4	1	2	-
STEC760	Goat	Shandong	2017	O50	NT	1	2	-
STEC761	Goat	Shandong	2017	O19	H4	1	2	-
STEC762	Goat	Shandong	2017	O74	H4	1	2	-
STEC763	Goat	Shandong	2017	O74	H4	1	2	-
STEC764	Beef cattle	Shandong	2017	O38	H4	1	2	-
STEC765	Goat	Shandong	2017	O71	H19	1	2	-
STEC766	Goat	Shandong	2017	O7	H19	1	2	-
STEC767	Goat	Shandong	2017	O53	H4	1	2	-
STEC768	Goat	Shandong	2017	O23	H4	1	2	-
STEC769	Goat	Shandong	2017	O38	H4	1	2	-
STEC770	Goat	Shandong	2017	O18	H25	1	-	-
STEC771	Goat	Shandong	2017	O57	H25	1	-	-
STEC772	Goat	Shandong	2017	O90	H19	1	-	-
STEC773	Goat	Shandong	2017	O74	H19	1	-	-
STEC774	Goat	Shandong	2017	O74	H8	1	-	-
STEC788	Water	Shandong	2017	NT	NT	-	2	-
STEC789	Goat	Shandong	2017	NT	NT	1	2	-
STEC790	Beef cattle	Shandong	2017	NT	NT	1a	2a	+
STEC791	Goat	Shandong	2017	O19	H19	1	-	-
STEC801	Diarrheal patient	Beijing	2018	NT	H7	1a	2c	+
